# Growth and Productivity Response of Hybrid Rice to Application of Animal Manures, Plant Residues and Phosphorus

**DOI:** 10.3389/fpls.2016.01440

**Published:** 2016-10-18

**Authors:** Amanullah, Shams-ul-Tamraiz Khan, Asif Iqbal, Shah Fahad

**Affiliations:** ^1^Department of Agronomy, University of AgriculturePeshawar, Pakistan; ^2^National Key Laboratory of Crop Genetic Improvement, MOA Key Laboratory of Crop Ecophysiology and Farming System, College of Plant Science and Technology, Huazhong Agricultural UniversityWuhan, China

**Keywords:** animal manures, plant residues, phosphorus levels, hybrid rice, growth, yield components, grain yield, Northwest Pakistan

## Abstract

The objective of this research was to evaluate the impact of organic sources (animal manures vs. plant residues at the rate of 10 t ha^−1^ each) on the productivity of hybrid rice (*Oryza sativa* L.) production under different levels of phosphorus (0, 30, 60, and 90 kg P ha^−1^) fertilization. Two separate field experiments were conducted. In experiment (1), impact of three animal manures sources (cattle, sheep, and poultry manures) and P levels were studied along with one control plot (no animal manure and P applied) was investigated. In experiment (2), three plant residues sources (peach leaves, garlic residues, and wheat straw) and P levels were studied along with one control plot (no plant residues and P applied). Both the experiments were carried out on small land farmer field at District Swabi, Khyber Pakhtunkhwa Province (Northwest Pakistan) during summer 2015. The results revealed that in both experiments the control plot had significantly (*p* ≤ 0.05) less productivity than the average of all treated plots with organic sources and P level. The increase in P levels in both experiments (animal manure vs. plant residues) resulted in higher rice productivity (90 > 60 > 30 > 0 kg P ha^−1^). In the experiment under animal manures, application of poultry manure increased rice productivity as compared with sheep and cattle manures (poultry > sheep > cattle manures). In the experiment under plant residues, application of peach leaves or garlic residues had higher rice productivity than wheat straw (peach leaves = garlic residues > wheat straw). On average, rice grown under animal manures produced about 20% higher grain yield than rice grown under crop residues. We conclude from this study that application of 90 kg P ha^−1^ along with combined application of animal manures, especially poultry manure increases rice productivity. Also, the use of either garlic residues or peach leaves, never applied before as organic manures, can increase crop productivity and will help in degraded soil for sustainable soil management.

## Introduction

Rice (*Oryza sativa* L.) is one of the most important staple foods for nearly half of the world's population, most of them living in developing countries. Rice provides 50–60% of the calories to 2.7 billion people (Belder et al., 2004; Metwally et al., 2011). Rice occupies about 11% of world's agricultural land and ranks second in terms of cultivated area (Tumrani et al., 2015). Rice is the second most widely consumed cereal in the world next to wheat. It is the staple food for two thirds of the world's population. Rice protein, though small in amount, is of high nutritional value (Chaudhary and Tran, 2001). In Pakistan, rice is the third largest crop after wheat (*Triticum aestivum* L.) and cotton (*Gossypium hirsutum* L.) on the basis of cultivated area and ranked second after wheat on production basis. It accounts for 5.9% of value added in agriculture and 1.3% of Pakistan's gross domestic product (Federal Bureau of Statistics, 2008–2009). Rice production in Pakistan occupies an area of 2.96 million hectares with a total production of 6.95 million tons and average yield of 2.35 t ha^−1^ (Federal Bureau of Statistics, 2008–2009). The average yield of rice in Pakistan has increased as a result of many research activities by more than 2% per year. This is still far less than other leading rice growing countries (Ito et al., 1989; Amanullah and Hidayatullah, 2016; Amanullah and Inamullah, 2016a).

One major reason for low rice yield in Pakistan is imbalanced nutrient application (Amanullah and Hidayatullah, 2016; Amanullah and Inamullah, 2016a,b). Integrated use of chemical fertilizers along with organic manure has been widely recommended for sustaining agricultural production in Northwest Pakistan (Amanullah and Inamullah, 2015; Amanullah and Khan, 2015; Amanullah and Khalid, 2016). Improved management practices such as incorporation of crop residue, animal manures along with chemical fertilizers application increase soil organic carbon and improve crop productivity (Six et al., 2002; Vanden et al., 2003; Rakshit et al., 2008; Amanullah and Hidayatullah, 2016; West and Post, 2002). Management of organic manures and chemical fertilizers account for 50–60% of the increase in field crop productivity (Dipa, 2006). Studies from different parts of the world have suggested that application of animal manures increase the yield of various crops (Olayinka, 1996; Olayinka et al., 1998; Ismail et al., 1990; Adepetu et al., 2005; Ayoola and Makinde, 2008; Hidayatullah et al., 2013; Iqbal et al., 2015; Amanullah and Khalid, 2016). Animal manure can serve as an important source for improving crop production and restoring essential nutrients depleted due to intensive cropping practices (Obi and Ebo, 1995; Sharpley and Smith, 1995; Bahman and James, 1999; Ano and Agwu, 2005; Materechera, 2010). Incorporation of crop residues also can improve soil fertility and increase crop yield (Singh et al., 2000; Prassad et al., 2002; Yadvinder et al., 2005; Bi et al., 2009; Olk et al., 2009; Kim et al., 2011; Huang et al., 2012). Nutrients from organic manures (animal manures and crop residues) are released more slowly and are stored for a longer time in the soil, thereby ensuring a long residual effect (Sharma et al., 1991; Ge et al., 2010; Hidayatullah et al., 2013; Amanullah and Khalid, 2016). However, application of large quantities of plant residues under anaerobic soil conditions may lead to the accumulation of phytotoxic substances (e.g., reducing substances and organic acids) that can inhibit rice growth and reduce grain yield (Bijay-Singh et al., 2008). Recently, Amanullah and Hidayatullah (2016) found reduction in hybrid rice productivity with application of wheat straw as compared with animal manures (poultry, cattle, and sheep manures) and plant residues (onion and berseem residues).

Phosphorus is the second most required plant nutrient for field crops. It plays a vital role in several physiological processes of plants (Mandal and Khan, 1972; Singh and Sale, 2000; Chang et al., 2007; Amanullah, 2011; Vahed et al., 2012). It is always inadequate under rice based system in Northwest Pakistan to meet the requirement of rice plants (Amanullah and Inamullah, 2016a,b). Recent research conducted on different field crops suggested that low organic matter content (Materechera, 2010; Verhulst et al., 2011; Enujeke, 2013; Hidaytaullah and Amanulla, 2015; Iqbal et al., 2015; Amanullah and Hidayatullah, 2016; Amanullah and Khalid, 2016) and low availability of phosphorus (Garg and Bahla, 2008; Alam et al., 2009; Bakhsh et al., 2012; Rasavel and Ravichandran, 2013; Yoseftabar, 2013; Amanullah and Khan, 2015; Amanullah and Inamullah, 2016a,b; Amanullah and Khalid, 2016) reduce crop productivity all over the world. Integrated use of mineral phosphorus along with animal manures or plant residues can improve rice growth and yield. We designed two field experiments to find out the best combination of inorganic phosphorus fertilization level with locally available sources of animal manures or plant residues for improving growth and yield of hybrid rice.

## Materials and methods

### Site description

Field experiments were conducted to evaluate the impact of different organic sources, each applied at the rate of 10 t ha^−1^, on the growth and yield of rice hybrid (*Oryza sativa* L., Almas) under different levels of phosphorus (0, 30, 60, and 90 kg P ha^−1^) fertilization along with one control plot in each experiment (no organic source and no P applied). Two field experiments were conducted in District Swabi Village Marghuz during summer-2015. Marghuz Village is at an altitude of 319 m (Latitude = 34.07, Longitude = 72.53). The village has a population of approximately 25,000, and consists of two parts: Marghuz Yara Khel and Marghuz Aka Khel (Wikipedia.org). District Swabi is situated at a distance of 100 km north-east of Peshawar, the provincial capital of Khyber Pakhtunkhwa. Soil texture is alluvium clay loam, low in organic matter (0.87%), extractable phosphorus (6.57 mg kg^−1^), exchangeable potassium (121 mg kg^−1^), and alkaline (pH 8.2) and is calcareous in nature (Amanullah et al., 2009). The climate of the Swabi is semiarid where the mean annual rainfall is 300–500 mm with 60–70% occurring in summer and 30–40% in winter (Amanullah et al., 2010a).

### Experimentation

Two field experiments were conducted in a simple randomized complete block design having three replications in each experiment. In the first experiment, the impact of three different animal manures (cattle, sheep, and poultry manures) in combination with three P levels along with one control plot (no animal manure and P applied) was studied (3 × 3 = 9 + 1 = 10 treatments per replication, randomly assigned). In the second experiment, the impact of three different plant residues (peach leaves, garlic residues, and wheat straw) in combination with three P levels along with one control plot (no plant residues and P applied) was studied (3 × 3 = 9 + 1 = 10 treatments per replication, randomly assigned). Details of the statistical analysis are in Table 1A for the first experiment and Table 1B for the second experiment.

**Table 1A:** **Analysis of variance for grain yield (kg ha^**−1**^) of rice hybrid as affected by phosphorus levels and animal manures (experiment one)**.

**Source of variance**	***DF***	***SS***	***MS***	***F*-Cal**	**Significance**
Replications	2	3861.667	1930.833	0.026362	ns
Treatments	{9}	7,099,270	788,807.8	10.76989	^***^
Phosphorus (P)	(2)	1,119,289	559,644.4	7.641038	^**^
Animal Manures (AM)	(2)	573,800	286,900	3.917154	^*^
AM × P	(4)	206,177.8	51,544.44	0.703756	Ns
Control vs. Rest	(1)	5,200,003	5,200,003	70.99761	^***^
Error-1	18	1,318,355	73,241.94		
Total	29	8,421,487			

*, **, and *** *indicates that data is significant at 5, 1, and 0.1% level of probability, respectively. The word ns stand for the non-significant data at 5% level of probability*.

*( ) stands for splits of {9} degrees of freedom (DF)*.

**Table 1B:** **Analysis of variance for grain yield (kg ha^**−1**^) of rice hybrid as affected by phosphorus levels and plant residues (experiment two)**.

**Source of variance**	***DF***	***SS***	***MS***	***F*-Cal**	**Significance**
Replications	2	49,040	24,520	0.607912	ns
Treatments	{9}	16,080,803	1,786,756	44.29811	^***^
Phosphorus (P)	(2)	10,720,096	5,360,048	132.8889	^***^
Plant Residue (PR)	(2)	868,585.2	434,292.6	10.76719	^***^
PR × P	(4)	367,837	91,959.26	2.279898	ns
Control vs. Rest	(1)	4,124,285	4,124,285	102.2512	^***^
Error-1	18	726,026.7	40,334.81		
Total	29	16,855,870			

*** *indicates that data is significant at 0.1% level of probability. The word “ns” represent non-significant outcomes at 5% level of probability*.

*( ) stands for splits of {9} degrees of freedom (DF)*.

A sub-plot size of 9 m^2^ (3 × 3 m) with hill to hill distance of 25 cm apart was used. A uniform dose of 120 kg N ha^−1^ as urea was applied to all treatments. The required nitrogen was applied in two equal splits: 50% each at transplanting and 30 days after transplanting, and P in the form of triple super phosphate (46% P_2_O_5_) was applied just before transplanting in a single dose at either 30, 60, or 90 kg P ha^−1^ according to Amanullah and Inamullah (2016a). The animal manures or plant residues (10 t ha^−1^ each) were applied to their respective plots 1 month before transplanting (Amanullah and Hidayatullah, 2016). All subplots were separated by about 30-cm ridges to stop movement of water/nutrients among different treatments (Amanullah and Inamullah, 2016a). Water to each treatment was separately applied from water channel. All other agronomic practices were carried out uniformly for all the experimental units throughout the rice growing season.

### Data recording and statistical analysis

Data on various measures of phenological development, days to panicle initiation and days to physiological maturity (Inamullah, 2014; Hidaytullah, 2015) plant height (Hidaytaullah and Amanulla, 2015); leaf characteristics, number of leaves tiller^−1^, leaf area hill^−1^, and leaf area index (Amanullah et al., 2016a); yield components (number of tillers hill^−1^, number of seeds panicle^−1^, 1000 grains weight) and grain yield (Amanullah and Hidayatullah, 2016); biological yield, straw yield, and harvest index (Amanullah and Inamullah, 2016a) were recorded. Data on all parameters of rice hybrid were subjected to analysis of variance (ANOVA) according to the methods described for simple randomized complete block design (Steel et al., 1996), and means between treatments were compared using LSD (least significant difference) test (*p* < 0.05). In both experiments the interactions between phosphorus and animal manures (experiment one), and between phosphorus and plant residues (experiment two) had no significant (*P* ≤ 0.05) effect on any parameters; therefore, it was excluded from the final model.

## Results

### Experiment one (phosphorus and animal manures)

#### Growth

Application of poultry manure enhanced days to panicle initiation, produced the tallest plants with more tillers and higher leaf area per hill (Table 2). There were no significant differences among manure treatments for number of leaves per tiller and leaf area index at panicle initiation (Table 2). Similarly, there were no significant differences among manure treatments for number of leaves per tiller at physiological maturity (Table 3). At physiological maturity, application of poultry manure reduced the days to physiological maturity, produced the tallest plants with more tillers and higher leaf area per hill that resulted in higher leaf area index (Table 3). Similarly, higher rates of P addition reduced the number of days to panicle ignition and days to physiological maturity, produced taller plants with more tillers per hill, more leaves per tiller with higher leaf area per hill and leaf area index at panicle initiation (Table 2) and physiological maturity (Table 3). Control plots took the longest to reach panicle initiation and physiological maturity, produced smallest plants with fewer tillers per hill, fewer leaves per tiller, lower leaf area per hill and lower leaf area index at both growth stages (Tables 2, 3).

**Table 2:** **Days to panicle initiation (PI), plant height (cm), number of tillers hill^**−1**^, number of leaves tiller^**−1**^, leaf area hill^**−1**^ (cm^**2**^), and leaf area index at panicle initiation of rice hybrid as affected by phosphorus levels (P) and animal manures (AM)**.

**Animal manures (10 t ha^−1^)**	**Days to panicle initiation**	**Plant height (cm)**	**Number of tillers hill^−1^**	**Number of leaves tiller^−1^**	**Leaf area hill^−1^ (cm^2^)**	**Leaf area index**
Cattle	57.8	93.6	14.6	6.2	2404	3.7
Sheep	57.8	94.6	14.8	6.2	2493	3.8
Poultry	56.9	95.4	15.0	6.4	2688	4.1
**LSD_0.05_**	**0.7**	**1.4**	**0.4**	**ns**	**337**	**ns**
**Phosphorus (kg ha^−1^)**						
30	59.1	91.6	14.0	5.9	2019	3.1
60	56.9	94.4	14.6	6.2	2480	3.8
90	56.4	97.6	15.8	6.8	3086	4.7
**LSD_0.05_**	**0.7**	**1.4**	**0.4**	**0.6**	**337**	**0.5**
Control	61.0a	86.3b	12.3b	5.7a	1203b	1.9b
Rest	57.5b	94.5a	14.8a	6.3a	2528a	3.9a
Interaction (AM × P)	Ns	ns	ns	ns	ns	ns

*Mean followed by different letters in the same category are significantly different using LSD-test (P ≤ 0.05). Where ns stands for non-significant data at P ≤ 0.05*.

**Table 3:** **Days to physiological maturity (PM), plant height (cm), number of tillers hill^**−1**^, number of leaves tiller^**−1**^, leaf area hill^**−1**^ (cm^**2**^), and leaf area index at physiological maturity of rice hybrid as affected by phosphorus (P) and animal manures (AM)**.

**Animal manures (10 t ha^−1^)**	**Days to physiological maturity**	**Plant height (cm)**	**Number of tillers hill^−1^**	**Number of leaves tiller^−1^**	**Leaf area hill^−1^ (cm^2^)**	**Leaf area index**
Cattle	102.1	101.7	22.1	4.8	1284	2.1
Sheep	101.6	104.3	22.1	4.9	1371	2.2
Poultry	101.1	107.3	23.1	5.2	1542	2.5
**LSD_0.05_**	**0.5**	**1.2**	**0.8**	**ns**	**160**	**0.3**
**Phosphorus (kg ha^−1^)**						
30	102.9	101.7	20.3	4.4	1034	1.7
60	101.4	104.3	22.4	5.0	1381	2.2
90	100.4	107.3	24.6	5.4	1782	2.9
**LSD_0.05_**	**0.5**	**1.2**	**0.8**	**0.5**	**160**	**0.3**
Control	104.3a	96.3b	16.0b	4.3b	872b	1.4b
Rest	101.6b	104.4a	22.4a	5.0a	1399a	2.2a
Interaction (AM × P)	Ns	ns	ns	ns	ns	ns

*Mean followed by different letters in the same category are significantly different using LSD-test (P ≤ 0.05). Where ns stands for non-significant data at P ≤ 0.05*.

#### Yield and yield components

There were generally no significant differences among manure treatments for straw yield and harvest index of rice (Table 4). Number of seeds per panicle, 1000 grains weight, biological and grain yield were all highest in plots under poultry manure as compared with cattle and sheep manures (Table 4). Increase in P rate increased seeds per panicle, 1000 grains weight, straw, biological, and grain yield. As with growth measures, yield and yield components were lowest in the control (Table 4).

**Table 4:** **Number of seeds panicle^**−1**^, 1000 grains weight (g), biological yield (kg ha^**−1**^), straw yield (kg ha^**−1**^), grain yield (kg ha^**−1**^), and harvest index (%) of rice hybrid as affected by phosphorus (P) and animal manures (AM)**.

**Animal manures (10 t ha^−1^)**	**Number of seeds panicle^−1^**	**Thousand grains weight (g)**	**Biological yield (kg ha^−1^)**	**Straw yield (kg ha^−1^)**	**Grain yield (kg ha^−1^)**	**Harvest index (%)**
Cattle	116.2	33.6	13,847	7046	6801	49.2
Sheep	116.8	34.6	14,310	7422	6888	48.2
Poultry	119.8	35.8	14,457	7313	7144	49.5
**LSD_0.05_**	**2.2**	**1.5**	**385**	**ns**	**268**	**ns**
**Phosphorus (kg ha^−1^)**						
30	108.6	32.4	13,542	6837	6704	49.5
60	117.6	34.8	14,261	7334	6927	48.6
90	126.7	36.7	14,811	7609	7202	48.6
**LSD_0.05_**	**2.2**	**1.5**	**385**	**468**	**268**	**ns**
Control	93.0b	28.0b	12,188b	6631b	5557b	45.9b
Rest	117.6a	34.6a	14,205a	7260a	6944a	48.9a
Interaction (AM × P)	ns	ns	ns	ns	ns	ns

*Mean followed by different letters in the same category are significantly different using LSD-test (P ≤ 0.05). Where ns stands for non-significant data at P ≤ 0.05*.

### Experiment two (phosphorus and plant residues)

#### Growth

Compared to wheat straw, application of garlic residues and peach leaves enhanced days to panicle initiation, produced the tallest plants with more tillers per hill and more leaves per plant that resulted in higher leaf area per hill and leaf area index at panicle initiation (Table 5). At physiological maturity, application of garlic residues and peach leaves reduced days to physiological maturity, produced the tallest plants with more leaves per plant, more tillers and higher leaf area per hill that resulted in higher leaf area index (Table 6). Like experiment one, higher rates of P addition reduced the number of days to panicle initiation and days to physiological maturity, produced taller plants with more tillers per hill, more leaves per tiller with higher leaf area per hill and leaf area index at panicle initiation and physiological maturity (Tables 5, 6). Control plots took the longest to reach panicle initiation and physiological maturity, produced smallest plants with fewer tillers per hill, fewer leaves per tiller, lower leaf area per hill and lower leaf area index at both growth stages (Tables 5, 6).

**Table 5:** **Days to panicle initiation (PI), plant height (cm), number of tillers hill^**−1**^, number of leaves tiller^**−1**^, leaf area hill^**−1**^ (cm^**2**^), and leaf area index at panicle initiation of rice hybrid as affected by phosphorus (P) and plant residues (PR)**.

**Plant residue (10 t ha^−1^)**	**Days to panicle initiation**	**Plant height (cm)**	**Number of tillers hill^−1^**	**Number of leaves tiller^−1^**	**Leaf area hill^−1^ (cm^2^)**	**Leaf area index**
Wheat straw	56.2	92.4	13.3	5.2	1501	2.4
Garlic residues	55.2	94.1	14.1	5.8	1993	3.2
Peach leaves	55.2	92.4	14.2	5.8	1914	3.1
**LSD_0.05_**	**0.6**	**1.1**	**0.5**	**0.5**	**206**	**0.3**
**Phosphorus (kg ha^−1^)**						
30	56.1	90.6	12.8	5.1	1230	2.0
60	55.7	93.2	14.0	5.7	1803	2.9
90	54.9	95.2	14.9	6.0	2375	3.8
**LSD_0.05_**	**0.6**	**1.1**	**0.5**	**0.5**	**206**	**0.3**
Control	59.3a	85.3b	11.7b	5.0b	978b	1.4b
Rest	55.6b	93.0a	13.9a	5.6a	1803a	2.9a
Interaction (PR × P)	ns	ns	ns	ns	ns	ns

*Mean followed by different letters in the same category are significantly different using LSD-test (P ≤ 0.05). Where ns stands for non-significant data at P ≤ 0.05*.

**Table 6:** **Days to physiological maturity (PM), plant height (cm) at PM, number of tillers hill^**−1**^ at PM, number of leaves tiller^**−1**^ at PM, leaf area hill^**−1**^ (cm^**2**^) at PM, and leaf area index at PM of rice hybrid as affected by phosphorus (P) and plant residues (PR)**.

**Plant residues (10 t ha^−1^)**	**Days to physiological maturity**	**Plant height (cm)**	**Number of tillers hill^−1^**	**Number of leaves tiller^−1^**	**Leaf area hill^−1^ (cm^2^)**	**Leaf area index**
Wheat straw	103.0	102.4	11.4	4.4	1266	2.0
Garlic residues	102.0	104.1	12.3	4.9	1658	2.7
Peach leaves	101.2	102.9	11.9	4.8	1517	2.4
**LSD_0.05_**	**0.6**	**1.1**	**1.0**	**0.3**	**197**	**0.3**
**Phosphorus (kg ha^−1^)**						
30	103.2	100.6	11.1	4.0	961	1.5
60	101.7	103.3	11.8	4.8	1496	2.4
90	101.3	105.6	12.7	5.3	1984	3.2
**LSD_0.05_**	**0.6**	**1.1**	**1.0**	**0.3**	**197**	**0.3**
Control	105.3a	95.3b	10.0b	4.0b	779b	1.2b
Rest	102.1b	103.1a	11.9a	4.7a	1480a	2.4a
Interaction (PR × P)	ns	ns	ns	ns	ns	ns

*Mean followed by different letters in the same category are significantly different using LSD-test (P ≤ 0.05). Where ns stands for non-significant data at P ≤ 0.05*.

#### Yield and yield components

There were generally no significant differences among plant residue treatments for harvest index of rice (Table 7). Number of seeds per panicle, 1000 grains weight, biological and grain yield increased significantly with application of either peach leaves or garlic residues as compared with wheat straw (Table 7). Like experiment one, increase in P rate increased seeds per panicle, 1000 grains weight, straw, biological and grain yield as well as harvest index. As with growth measures, yield and yield components were lowest in the control (Table 7).

**Table 7:** **Number of seeds panicle^**−1**^, 1000 grains weight (g), biological yield (kg ha^**−1**^), straw yield (kg ha^**−1**^), grain yield (kg ha^**−1**^), and harvest index (%) of rice hybrid as affected by phosphorus (P) and plant residues (PR)**.

**Plant residue (10 t ha^−1^)**	**Number of seeds panicle^−1^**	**Thousand grains weight (g)**	**Biological yield (kg ha^−1^)**	**Straw yield (kg ha^−1^)**	**Grain yield (kg ha^−1^)**	**Harvest index (%)**
Wheat straw	121.2	32.2	11,922	6633	5289	44.2
Garlic residues	124.2	33.8	12,478	6987	5491	43.9
Peach leaves	123.9	32.2	12,778	7050	5728	44.7
**LSD_0.05_**	**2.5**	**1.1**	**458**	**520**	**199**	**ns**
**Phosphorus (kg ha^−1^)**						
30	115.7	30.7	11,233	6583	4650	41.4
60	122.3	33.3	12,689	6984	5704	45.1
90	131.3	34.2	13,256	7102	6153	46.5
**LSD_0.05_**	**2.5**	**1.1**	**458**	**520**	**199**	**2.5**
Control	102.7b	26.7b	10,500b	6233b	4267b	40.7b
Rest	123.1a	32.7a	12,393a	6890a	5503a	44.3a
Interaction (PR × P)	Ns	ns	ns	ns	ns	ns

*Mean followed by different letters in the same category are significantly different using LSD-test (P ≤ 0.05). Where ns stands for non-significant data at P ≤ 0.05*.

## Discussion

### Phosphorus

The increased rate of phenological development with increasing P fertilization has been seen in other studies on different crops (Amanullah and Khan, 2010; Khalil et al., 2010; Amanullah et al., 2010b, 2014; Amanullah, 2011; Amanullah and Khalid, 2015). As P deficiency can decrease both root and shoot development (Amanullah and Stewart, 2013) and phosphorus uptake (Amanullah and Inamullah, 2016b), which can cause a delay in phenological development (Amanullah et al., 2014; Amanullah and Khalid, 2015; Amanullah et al., 2016b). Increasing P level also improves plant growth parameters due to an increase in leaf photosynthetic rate and formation of more photosynthates (Sarika et al., 2006; Amanullah et al., 2013). Increase in growth parameters in different crops while increasing P rate is earlier reported in many studies from different parts of the world (Akinrinde and Gaizer, 2006; Fageria et al., 2006; Opala et al., 2009; Rast et al., 2010; Rasavel and Ravichandran, 2013). Fageria et al. (2003) and Fageria and Baligar (2005) reported that tillering capacity in rice was considerably affected by P application. The increase in LAI in this study with increase in P level probably may be due the role of P in promoting plant growth and development, increase in number of tillering, and root development in rice (Amanullah, 2011). The increase in yield and yield components, and harvest index while increasing P level may be due to the higher availability and uptake of phosphorus (Amanullah and Inamullah, 2016b) and zinc (Inamullah, 2014). According to Rahman et al. (2011), higher P level increases the availability of P and Zn that had considerably helpful effect on growth, yield and yield contributing parameters (Hao et al., 2009). The results are also in line with Begum et al. (2002) who reported the highest bearing tillers hill^−1^ from seedling raised with proper nutrients availability. According to Makino (2011), the higher grain yield may be attributed due to better growth with higher nutrient availability (Akinrinde and Gaizer, 2006; Fageria et al., 2006; Rast et al., 2010; Rasavel and Ravichandran, 2013; Opala et al., 2009) and higher photosynthetic rate of the plants (Sarika et al., 2006) and more photosynthate partitioning into the reproductive parts (Baruah et al., 2012; Amanullah and Inamullah, 2016a). Increase in number of tillers hill^−1^, panicle length, grains panicle^−1^ and panicles m^−2^ was noticed with application of P over control (Bakhsh et al., 2012; Rasavel and Ravichandran, 2013). Fageria (2007) reported an increase of 80% in biological yield and 180% in grain yield of rice with the application of higher P rate of 131 kg P ha^−1^. Rasavel and Ravichandran (2013) reported the highest grain and straw yields with only 50 kg P_2_O_5_ ha^−1^ application. Yoseftabar (2013), on the other hand, recorded maximum grain and biological yield with 90 kg P ha^−1^. Recently, Amanullah and Inamullah (2016a) suggested that increase in P level increases dry matter partitioning into the panicles of rice, increasing panicle weight and harvest index in different rice genotypes. Mafi et al. (2013) also reported that increasing P level increased yield and harvest index in rice.

### Organic sources

The results of our study also demonstrated that application of different animal manures (experiment one) and plant residues (experiment two) significantly improved phenological development, growth parameters, yield and yield components of hybrid rice. The differences in the C:N ratios of different organic sources (Amanullah and Hidayatullah, 2016) may have influenced the availability and uptake of nutrients, especially phosphorus, by the plants that affected phenology, growth, and yield. Hidaytullah (2015) found earlier phenological development and better growth for hybrid rice (Pukhraj) with application of poultry manure due to its lower C:N ratio than other organic sources used (sheep manure, cattle manure, onion leaves, berseem residues, and wheat straw). Variation in growth parameters while using organic manure was reported earlier by Babu et al. (2001) and Rani et al. (2001). Recently, (Hidaytullah, 2015) and Amanullah and Khalid (2015), and Amanullah and Hidayatullah (2016) revealed that application of poultry manure (with lower C:N ratio) increased growth and yield of hybrid rice because of the higher nitrogen availability (Hidaytullah, 2015). Mohandas et al. (2008) and Enujeke (2013) also reported that application of poultry manure increased plant growth and yield because more nutrients were made readily available and easily absorbable by receiving plants leading to faster growth and development. Poultry manures application also might have improved soil quality to enhance the yield components and grain yield of rice (Larson and Clapp, 1984; Fagimi and Odebode, 2007). According to Ewulo et al. (2008), addition of poultry manure (50 t ha^−1^) reduced soil bulk density (1.11 g/cc) & soil pH (6.2), and increased soil organic matter (2.77%), nitrogen (0.31%), phosphorus (32.6 mg/kg) and soil moisture (17.2%) as compared with control (no poultry manure applied) with higher soil bulk density (1.43 g/cc) & soil pH (7.0), and lower soil organic matter content (1.40%), nitrogen (0.09%), phosphorus (10.6 mg/kg), and soil moisture content (11.9%). The improvement in soil quality is primarily attributable to improvement in soil organic matter content. Aluko and Oyedele (2005) reported that increase in soil moisture content with poultry manure is attributable to its mulching effect which improved moisture retention and water acceptance as a result of improved soil structure and macro porosity. Amanullah and Hidayatullah (2016) found that application of animal based manures (poultry, sheep, and cattle) were far better in terms of higher yield and yield components of rice as compared with plant residues (berseem, onion, and wheat). Wheat straw, on the other hand, had negative influence on yield and yield components in hybrid rice (Amanullah and Hidayatullah, 2016). The negative impact of plant residues on rice under anaerobic conditions was earlier reported by Yadvinder et al. (2005) and Olk et al. (2009). The decrease in harvest index under plant residues was due to the poor P content in plant residues, and also plant residue P retention in soil was low (Yadvinder et al., 2005; Amanullah and Inamullah, 2016a). Hossaen et al. (2011), Hidayatullah et al. (2013), and Amanullah and Khan (2015) reported that application of organic manures increase yield and harvest index over control. Amanullah and Stewart (2015) confirmed that leaf area per plant, leaf area expansion rate, specific leaf area, leaf area ratio, plant height, stem elongation rate, root length, number of roots per plant, number of tillers per plant, absolute growth rate and crop growth rate increased significantly in both wheat and rye crops grown under organic soils as compared with inorganic soils at different growth stages.

## Conclusion

Poultry manure was a better organic source for improving growth and yield of hybrid rice as compared to sheep and cattle manures. Among the plant residues both garlic residue and peach leaves were found more beneficial in terms of better growth and higher yield over wheat straw. Higher phosphorus level (90 kg P ha^−1^) was found more beneficial in terms of better growth, higher yield and yield components under both experiments. We recommend a combination of inorganic P with organic sources to maximize not only growth and yield but also soil improvement for hybrid rice production in Northwest Pakistan.

## Author contributions

A: Designed the research project, supervised the research work, and wrote the final manuscript. STK: Conducted the field experiments, took data, and wrote the report for his degree requirement. AI: Helped in the statistical analysis of data, made tables, and downloaded related literature. SF: Downloaded literature and helped in the revision and improved the quality of the manuscript.

### Conflict of interest statement

The authors declare that the research was conducted in the absence of any commercial or financial relationships that could be construed as a potential conflict of interest.
